# First Isolation and Clinical Case of *Brevundimonas* *diminuta* in a Newborn with Low Birth Weight, in Democratic Republic of Congo: A Case Report

**DOI:** 10.3390/medicina57111227

**Published:** 2021-11-11

**Authors:** David Lupande-Mwenebitu, Raphael Kavul Tshiyongo, Octavie Lunguya-Metila, Jean-Philippe Lavigne, Jean-Marc Rolain, Seydina M. Diene

**Affiliations:** 1 Faculté de Pharmacie, Aix Marseille Université, IRD, APHM, MEPHI, IHU Méditerranée Infection, 19-21 Boulevard Jean Moulin, CEDEX 05, 13385 Marseille, France; lupande2000@gmail.com; 2 Hôpital Provincial Général de Référence de Bukavu, Université Catholique de Bukavu (UCB), Bukavu 285, Democratic Republic of the Congo; raphaelkavul@gmail.com; 3 Service de Microbiologie, Cliniques Universitaires de Kinshasa, Kinshasa 127, Democratic Republic of the Congo; octmetila@yahoo.fr; 4 VBIC, INSERM U1047, Service de Microbiologie et Hygiène Hospitalière, CHU Nîmes, Université de Montpellier, 30000 Nîmes, France; jean.philippe.lavigne@chu-nimes.fr

**Keywords:** *Brevundimonas diminuta*, omphalitis, low birth weight, nosocomial infection

## Abstract

*Brevundimonas diminuta* is rarely described in clinical specimens, never at the umbilical stump. Most of the reported cases are in patients with underlying pathologies. We must integrate this microorganism in the etiological agents of nosocomial infections, but much remains to be understood about its virulence. We present a case of umbilical stump infection (omphalitis) caused by *B. diminuta*, in a preterm and hypotrophic new-born and discuss the diagnosis of this bacterium and its role as responsible of nosocomial neonatal infections.

## 1. Introduction

*Brevundimonas* species are aerobic Gram-negative, oxidase and catalase positive, non-fermenting rods 1 to 4 mm in length and 0.5 mm in width, belonging to the Alphaproteobacteria class and Caulobacteraceae family with a DNA G + C content of 65% to 68% [1]. Infections caused by *Brevundimonas* species are rare in humans and are mainly nosocomial bacteremia in immunocompromised patients [2]. *B. vesicularis*, *B. nasdae* and *B. diminuta* are the three species isolated from human infections, while the other 29 species have not yet been isolated in humans until now [3]. Only a few clinical cases of serious opportunistic infections, particularly in patients with compromised immunity, have been reported for *B. diminuta* [4]. All known species of *Brevundimonas* spp. show a strong resistance to most antibiotics used; but according to the Centers for Disease Control and Prevention (CDC), *B. vesicularis* would be uniformly susceptible to aminoglycosides and anti-pseudomonal penicillins but would be resistant to ampicillin and cephalosporins [5].

*B. diminuta* is ubiquitous, with worldwide distribution, isolated from several sites including water, soil and plants. It is also described that it survives in disinfectants [6]. In the literature, *B. diminuta* is reported in humans mainly in cancer patients. These infections are mainly bacteremia, intravascular catheter infections, urinary tract infections, pleural damage (a case of empyema) and keratitis secondary to wearing visibly contaminated lenses [7,8,9,10]. Rare cases of *Brevundimonas* spp. infections are described in Africa, but several studies have isolated it in the polluted environment as well as in urban tributaries in South Africa, Libya, Nigeria and Tunisia [11,12,13,14]. Here we describe the first clinical case of *B. diminuta* infection in a low-birth-weight newborn in Bukavu, Democratic Republic of the Congo.

## 2. Material and Methods

Bacterial identification and confirmation were performed using Microflex LT MALDI-TOF mass spectrometer (Bruker Daltonics, Bremen, Germany) after shipment of the isolate to the Institut Hospitalo-Universitaire (IHU) Mediterranee Infection of Marseille, France for further analysis. Antibiotics susceptibility testing (AST) to 16 antimicrobial agents (i.e., amikacin, ticarcillin, ticarcillin/clavulanic acid, ceftazidime, cefepime, rifampicin, ciprofloxacin, colistin sulfate, doxycycline, meropenem, fosfomycin, gentamicin, imipenem, nitrofurantoin, tazobactam/piperacillin, trimethoprim/sulfamethoxazole (i2a, Montpellier, France)) was determined by the disk diffusion method according to the European Committee on Antimicrobial Susceptibility Testing (EUCAST) guidelines as updated in January 2013 [15]. DNA extraction was processed before molecular analysis. Automated DNA extraction was performed on 200 µL of each isolate using a BioRobot^®^EZ1 Advanced XL instrument (QIAGEN, Hilden, Germany) according to the manufacturer’s instructions. All quantitative real-time PCR (qPCR) reactions were performed using a C1000 Touch™ Thermal Cycle (Bio-Rad, Hercules, CA, USA) with the ready-to-use reaction mix ROX qPCR Master according to the manufacturer’s recommendations. Negative control (single PCR mix and sterile H_2_O) and positive control template (Plasmid DNA extracted from a colony of *K. pneumoniae* Kp_nasey) were included in each qPCR experimental run. Results were considered positive accepted when the cycle threshold value of real-time PCR was ≤35.

## 3. Result

### 3.1. Case Presentation

Here, we report a case of a newborn (April 2019), low-weight preterm of 36 gestational weeks, delivered by planned cesarean section, indicated for recurrent maternal hypoglycemia in active hepatitis B. A multiparous mother, who had good obstetric history with previous uncomplicated vaginal delivery, was 34 years old, transferred from Internal Medicine unit to the maternity, and was admitted to hospital a few days earlier with hypoglycemia and a Glasgow Coma Scale 2. Her HIV serology and STORCH (Syphilis Toxiplasmosis Rubeola Cytomegalovirus and Herpesvirus) serology were unknown. Up on physical examination, the newborn had a birth weight of 2400 g, a height of 46 cm, a head circumference of 34 cm, with an Apgar score of 9 and 9 at 1 and 5 min, respectively. He had poor general condition with dyspnea (only few minutes after birth), the axillary temperature was 36 °C, a heart rate at 156 beats per minute and a respiratory rate at 68 breaths per minute. Based on these findings the admission diagnosis was possible neonatal infection in preterm delivery and hypotrophic conditions. Results from laboratory studies were as follows: complete blood count; white blood cells 7.8 × 109/L, 20.6% lymphocyte and 69.6% neutrophil, red blood cells 3.4 × 109/L, hemoglobin at 13.5 g/dL, hematocrit at 36.7% and Platelets at 32.8 × 109/L. CRP was less than 6 mg/L (normal range less than 6 mg/L), urea 15 mg/dL (normal range 10–25 mg/dL), creatinine 0.66 mg/dL (normal range 0.29–1.04 mg/dL) with a glomerular filtration rate of 23.6 mL/min/1.73 m^2^ and blood cultures sampled. The evolution was marked by the persistence of the symptomatology and the appearance of purulent secretions at the umbilical stump (omphalitis), swab was collected for microbiological cultures. The newborn was fed with breast milk then after, got debridate syrup 3 times one dose per day, clindamycin three times 10 mg per day and amikacin once 30 mg per day as a treatment. The blood cultures showed negative results after 5 days of incubation and the umbilical stump secretions culture yielded lactose negative colonies on Mac Conkey medium and small sized, non-hemolytic colonies on blood agar medium. Gram staining of the colonies showed Gram-negative rods. The oxidase and catalase tests were positive, but the preliminary identification using conventional biochemical tests as urea, indole, citrate and triple sugar iron (TSI) was unsuccessful. The identification of *B. diminuta* has been done using the MALDI-TOF MS once the isolate has been transmitted to Marseille.

### 3.2. Molecular Characterisation

**Figure 1:**
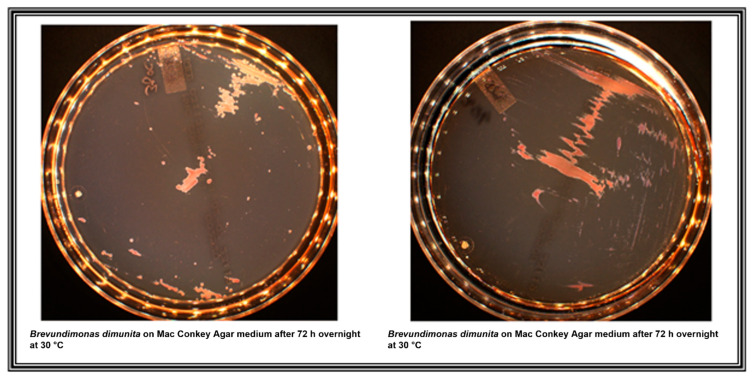
*B. diminuta* on Mac Conkey agar medium.

Bacterial identification of colonies from the Mac Conkey agar (Figure 1) using the MALDI-TOF reveals the presence of *B. diminuta* strain with score identification >3. The performed AST revealed that the *B. diminuta* isolate was susceptible to tazobactam/piperacillin, rifampicin, cefepime, meropenem, imipenem, amikacin, fosfomycin and doxycycline but was resistant to nitrofurantoin, ciprofloxacin, gentamicin, ceftazidime, ticarcillin, trimethoprim/sulfamethoxazole and ticarcillin clavulanate. According to the AST results, the qPCR amplification was used to confirm the presence of most common β-lactamases-encoding genes including *bla*_TEM_, *bla*_SHV_ and the absence of ESBL genes (i.e., *bla*_CTX-A_ and *bla*_CTX-B_ (*bla*_CTX-M_ clusters A and B)) as described previously [16]. After a few days of starting the treatment, the newborn began to show significant improvement, with drying of umbilical stump secretions and was discharged, while a follow-up control proposed two weeks after.

## 4. Discussion

To our best knowledge, this is the first case report describing *B. diminuta* as a pathogen responsible for omphalitis, a rare and almost unknown pathology in developed countries. *B. diminuta* is less well known clinically than *B. vesicularis*, and very few clinicians have treated the infections it has caused, even though its first identification and description date back to 1954 and the reference genome comes from this same strain [8]. Probably because this bacterium is less virulent than other, but also its identification is not easy in low-income countries. It has been reported that most strains do not grow on MacConkey agar, which is not the case for our strain (Figure 1) [17]. Contrary to the other 2 species, *B. diminuta* can be found in a person without comorbidities, which has not yet been observed with the other species [4]. However, we isolated it from a preterm and hypotrophic new-born, whose immaturity of the immune system could have contributed to its occurrence.

Despite its natural resistance to colistin, our strain was susceptible to carbapenems (imipenem and meropenem), cyclins (doxycycline), and resistant to quinolones (ciprofloxacin). This gives the physician a wide choice of management, contrary to the resistance profile reported in other studies with quinolone resistance [18,19]. Moreover, *B. diminuta* as well as all other species are ubiquitous (environment), and resistant to disinfectants, and we could speculate that this is the probable source of the infection in our case, unfortunately the investigations had not been carried out, because brushing the umbilical stump is done with disinfectants (e.g., betadine) in common practice, but we were not be able to specify here what was exactly used for this newborn. Furthermore, this infection could also have originated from the child’s mother, but here again we could not find evidence of an earlier or ongoing infection in the mother’s medical history, of which *B. diminuta* was identified [20,21].

*Staphylococcus aureus*, *Streptococcus* sp. and Enterobacterial species are the common agents of omphalitis, rare cases with *Pseudomonas aeruginosa* have been described, this is the first time that omphalitis with *B. diminuta* is documented [22,23,24,25].

## 5. Conclusions

In conclusion, to prevent omphalitis, the entire health care team must work together to ensure the use of aseptic techniques during delivery and the maintenance of proper umbilical cord care afterwards. The umbilical stump should receive special attention and daily monitoring in a safe environment with appropriate care and techniques. We must also integrate *B. diminuta* whenever there is an omphalitis in the context of a nosocomial infection.

## Data Availability

All the data are available from the corresponding author upon reasonable request.
